# Is low-nicotine Marlboro snus really snus?

**DOI:** 10.1186/1477-7517-5-9

**Published:** 2008-02-27

**Authors:** Jonathan Foulds, Helena Furberg

**Affiliations:** 1 University of Medicine and Dentistry of New Jersey-School of Public Health, Tobacco Dependence Program, 317 George Street, Suite 210, New Brunswick, NJ 08901, USA; 2 University of North Carolina Department of Genetics, 115 Mason Farm Road, Campus Box #7264, Chapel Hill, NC 27599-7264, USA

## Abstract

Swedish snus is a medium/high nicotine delivery, low-nitrosamine moist smokeless tobacco product that has been estimated to be at least 90% less harmful than smoked tobacco. More men use snus than smoke cigarettes in Sweden, and a quarter of male former smokers quit by switching to snus. Leading multinational cigarette manufacturers have begun test-marketing snus-like products in the United States and other countries. The version of Philip Morris' Marlboro snus currently being marketed in the United States differs from Swedish snus in many ways; it has lower moisture content and pH, but most puzzling is its very low nicotine delivery. Philip Morris, the market-leader in United States cigarette sales, may have designed the product so that it does not satisfy nicotine cravings and fails to enable smokers to switch. In this paper we compare and contrast Swedish snus and Marlboro snus, and speculate as to why Philip Morris may have intentionally designed a product that delivers very low levels of nicotine. We recommend that Philip Morris cease using the term "snus" to refer to dry tobacco products with low nicotine delivery, so that the term be reserved for moist, low-toxin, medium/high nicotine delivery smokeless tobacco products that are qualitatively similar to the leading brands in Sweden.

## 1. Introduction

At the beginning of the twenty-first century, few tobacco control advocates outside of Sweden had heard of "snus," the form of low-nitrosamine moist snuff tobacco that is very popular in that country [1]. As of 2007, most of the major multinational tobacco companies have begun test-marketing their own brands of snus, using their leading cigarette brand names to market new snus products (e.g., Camel snus, Lucky Strike snus, Peter Stuyvesant snus). While increasing scientific evidence indicates that Swedish snus is not harmless but is less harmful to health than cigarettes [2-6], the public health community has observed the launch of these new snus products outside of Sweden with increasing apprehension [7-9].

Philip Morris USA (PM) started market testing its first snus product, called Taboka, in Indianapolis in 2006. Concern spiked when PM announced it would call its new brand, Marlboro Snus. Along with Coca-Cola, Marlboro is one of the top two "global megabrands" [10] and has approximately 40% of the cigarette market in the United States (U.S.). It would seem unlikely that the company would place its leading brand name on a product that it did not expect to succeed. However, data recently released by PM raises questions about the company's intentions and about the appropriateness of applying the term "snus" to this product.

In this paper we compare and contrast Swedish snus with the new PM smokeless tobacco product called Marlboro Snus, and speculate as to why PM has intentionally designed and marketed a smokeless tobacco product that delivers relatively low levels of nicotine.

## 2. Discussion

### What is Swedish Snus?

Swedish snus is an oral smokeless tobacco product that typically contains approximately 50% moisture. Its rather high pH (7.5–8.5) results in a high proportion of "free" or unbound nicotine, which facilitates nicotine absorption into the body [11]. A single 2 g dose of a leading brand of Swedish snus is placed underneath the upper lip (reducing salivation) and gives the user a boost in blood nicotine concentration of around 15 ng/ml within 30 minutes [12,13]. The relatively high nicotine delivery of Swedish snus is similar to a cigarette, and much higher than most existing nicotine replacement therapies including nicotine gum, lozenge, inhaler and nasal spray. Notably, Swedish snus is characterized by low concentrations of carcinogenic tobacco-specific nitrosamines (TSNAs) and other toxins relative to smokeless tobacco sold in the U.S. [6,11]. It is believed that the relatively low levels of toxins found in Swedish snus are due to the selection of air-cured tobacco already low in toxins, and the use of a pasteurization process which kills the microbes that otherwise contribute to the formation of carcinogenic TSNAs [6,11].

Swedish snus is therefore a moist snuff product characterized by low TSNA concentration but high nicotine delivery. It is believed that the low toxin levels, combined with the avoidance of smoke inhalation, are responsible for Swedish snus being associated with substantially lower health consequences than cigarette smoking [2-6]. Levy et al. estimated that the median mortality relative risk for low-nitrosamine smokeless tobacco was at least 90% lower than for cigarette smoking [5]. Over one quarter of male ex-smokers in Sweden reported they quit smoking by switching to snus [14,15]; it is possible that the relatively high nicotine delivery of Swedish snus makes the transition from cigarettes to snus more comfortable [14,16-20].

### Industry data on Marlboro Snus and other new snus products

The Life Sciences Research Office Inc. (LSRO) is a non-profit company located in Bethesda, Maryland USA. The LSRO claims to "help our clients digest and assimilate...information and turn it to their advantage". LSRO recently coordinated a series of meetings funded by PM and attended by representatives from various tobacco companies to discuss "Differentiating the Health Risks of Categories of Tobacco Products". The agenda, presentations, and brief minutes of these meetings are available to the public via the LSRO website and shed some light on tobacco industry strategy on harm reduction.

Of particular interest was the recent presentation by Michael T Fisher, PhD, of PM on "Snus smokeless tobacco products" [21]. The presentation highlighted four main differences between PM's new snus products and other traditional moist oral smokeless tobacco sold in the U.S. PM snus a) has a smaller portion size (0.23 g versus 1.5 g), b) is pasteurized rather than fermented, c) utilizes "flavor-film technology", and d) is dry, containing only 12% moisture versus 50% for traditional moist snuff.

In addition, Fisher's presentation compared the chemical constituents and 26 characteristics of 13 leading brands of U.S. loose snuff, 3 leading brands of loose Swedish snus, 9 leading brands of portion-packed Swedish snus and one brand of portion-packed U.S. snuff. Importantly, PM snus has characteristically low concentrations of TSNAs similar to Swedish snus. However, although PM snus contains a similar amount of nicotine per weight as Swedish snus, since its pH is below the bottom range for Swedish snus, the amount of "free" nicotine that can be absorbed from PM snus was well below the minimum of all the leading brands of U.S. and Swedish smokeless tobacco products. One slide in Fisher's presentation showed the blood nicotine levels found throughout the day in 26 subjects who either smoked normally or used PM snus. While smoking, these individuals had an afternoon blood nicotine concentration around 18 ng/ml (a relatively low concentration compared to other published data on afternoon blood nicotine in smokers [22]), whereas the afternoon level while using PM snus averaged under 4 ng/ml.

Fisher's data therefore reveal that PM snus, despite having low concentrations of toxins like Swedish snus, is different from its namesake in a number of important respects. PM snus is dry rather than moist and, given its low pH, delivers substantially lower levels of nicotine. Other differences include the addition of a "flavor strip", much smaller portion size, and manufacture in the U.S. rather than Sweden. These differences lead us to question, is PM Marlboro snus really snus at all? Of all the differences, the most important and puzzling difference is the very low nicotine delivery. Swedish snus has been perceived by some as having a public health benefit in Sweden and potentially in other countries by taking market share from cigarettes [6,14,23]. The high nicotine delivery of Swedish snus, which is similar to a cigarette, would appear to be critical to that effect.

### Why does Marlboro Snus deliver so little nicotine?

PM claims that their product design was based on an assessment of consumer acceptability. But PM must be aware that a tobacco product delivering minimal amounts of nicotine is of little use to most smokers. PM has previously tried to market an extremely low nicotine delivery cigarette (Next) that predictably failed in the marketplace (less than 0.2% market share) [24]. So why has PM chosen to test market two snus products, one which is available in four flavors, all with extremely low nicotine delivery?

One possibility is that these initial products are being tried in a few test markets simply to continue testing consumer taste preferences, and that eventually the product will evolve into a higher nicotine delivery product. Perhaps these new products are intentionally being designed as "graduation" products, from which starters will progress to higher nicotine delivery products.

Another more Machiavellian possibility is that PM is perfectly aware that smokers will not use a low nicotine smokeless product for long, just as auto manufacturers are aware that there isn't a large market for safer cars with a maximum speed of 30 miles per hour. In that case, we have to assume that the test-marketing of PM snus is intended to fail. What motive might PM have for such a bizarre use of its most famous brand? PM has, by far, the largest share of the U.S. cigarette market (50% including all brands); thus a consumer switching from cigarettes to smokeless is more likely to be leaving a PM cigarette brand than a brand of their competitors. Furthermore, PM has a high profit margin from its cigarettes that snus is unlikely to replicate. Thus, even smokers who switch from a PM cigarette brand to a PM snus brand will result in a lower profit margin. It is therefore not in PM's financial interest for snus to become as successful in the U.S. as it has been in Sweden. One way to avoid this is to market a product called snus without adequate delivery of the key ingredient, nicotine. Mass marketing of this product could potentially "vaccinate" U.S. smokers against switching to snus by teaching them that snus is an unsatisfying product with no nicotine "hit". Uniquely, PM is giving away coupons for free Marlboro snus tins and is even attaching free Marlboro snus samples to Marlboro cigarette packs.

Another motive may be perceived reduction of litigation risks. If snus were to become as popular in the U.S. as it has become among Swedish men (>50% of tobacco market among men [6]), it could make it much harder to defend smoking-caused lung-cancer law-suits, as it could then be reasonably argued by victims' lawyers that PM is a tobacco company that negligently and recklessly persisted in selling a needlessly harmful product when it had good evidence that it could stay in business selling a much less harmful tobacco product that does not cause lung cancer (i.e., snus). One way to avoid such an argument having merit in court is to demonstrate that snus is not an acceptable alternative to cigarettes in the U.S.

### Issues around snus use as harm reduction in the U.S

Concerns about the introduction of Swedish snus in the U.S. as a potential harm reduction product have been expressed in both scientific journals [7-9] and popular media [25], and center around two important issues. One issue focuses on the possibility that introducing a new, less harmful smokeless tobacco product will encourage use by young people. There is little doubt that snus will be used by young people just as cigarettes are. However, in Sweden, snus use appears to be a pathway from smoking, rather than being a gateway to smoking [14-17]. Furthermore, in northern Sweden, where snus use is most prevalent, only 3% of 25–34 year-old men are daily smokers, while 34% are daily snus users [18]. Thus, it appears that Swedish snus has replaced smoking for many young people in Sweden. This is the very age-group with the highest smoking prevalence in the United States who would have a lot to gain by quitting smoking [26].

Perhaps the biggest threat to public health from new low nitrosamine snus stems from the possibility that it may foster persistent dual tobacco use instead of smoking cessation. As restrictions on cigarette smoking increase, smokers who might otherwise have quit may instead use snus at times when they cannot smoke. A product delivering as little nicotine as PM snus will leave the smoker craving for a cigarette, possibly another part of PM's intention. Although dual cigarette and snus use is not the pattern emerging in Sweden [14,18], it is only recently that cigarette companies have simultaneously been marketing smokeless products in Sweden. Dual use of cigarettes and snus is certainly a valid concern that needs to be monitored closely.

## 3. Conclusion

Efforts to control tobacco-caused deaths and diseases continue to be hampered by inadequate regulatory control over tobacco products. The Royal College of Physicians (RCP) recently concluded that, "low nitrosamine smokeless tobacco products may have a positive role to play in a coordinated and regulated harm reduction strategy which maximizes public health benefit and protects against commercial market exploitation," (p230) [11]. It is unclear whether the form of FDA regulation currently proposed for the United States, and supported by PM, would embrace the model for tobacco harm reduction advocated by the RCP.

Right now in the United States, tobacco manufacturers can introduce new products, call them whatever they wish, and change product ingredients without adequate regulatory oversight or information for the public. It is clear that the status quo is unacceptable and simply allows the tobacco industry to confuse the public about the nature of its products [27]. If Marlboro snus continues to deliver very low levels of nicotine then it will likely not appeal to smokers as an alternative to cigarettes, just as PM's brand of cigarettes with very low nicotine delivery (Next) was unsuccessful in the marketplace. It is possible that PM intends for Marlboro snus to fail in the marketplace, and may even hope that the tobacco control lobby's criticism of snus will assist with its demise [7,28,29]. Criticism of snus by anti-tobacco groups may end up unwittingly supporting PM's efforts to maintain the cigarette-dominant status quo. In so doing, PM could prevent snus from challenging the dominance of cigarettes – a product that is at least ten times more harmful to health than snus [5], and the number one cause of premature death in the western world.

We recommend that PM cease using the term "snus" to refer to dry smokeless tobacco products with low nicotine delivery, so that the term be reserved for moist, low-toxin, medium/high nicotine delivery smokeless tobacco products that are qualitatively similar to the leading brands in Sweden.

## Abbreviations

PM = Philip Morris, U.S. = United States, TSNA = tobacco-specific nitrosamines, RCP = Royal College of Physicians.

## Competing interests

Jonathan Foulds has worked as a consultant and speaker for pharmaceutical companies involved in production of tobacco dependence treatment medications, as well as a variety of agencies involved in promoting health (e.g. W.H.O., N.I.H., etc). A number of these agencies have provided sponsorship funds for educational events conducted by the program he directs. The program he directs (Tobacco Dependence Program at UMDNJ-School of Public Health) conducts trainings and charges health professionals and their organizations for providing these. He has also worked as an expert witness in litigation, including for plaintiffs in law suits against tobacco companies. He has not received any funding from the tobacco industry other than deposition fees from defendants attorneys in litigation against the tobacco industry (i.e. while acting as a witness for the plaintiffs). He is paid for writing a regular column on a health website.

Helena Furberg is an Assistant Professor of Genetics at the University of North Carolina at Chapel Hill and has never received funding from the tobacco industry. She declares no conflict of interest.

## Authors' contributions

JF drafted the original manuscript and HF has been involved in revising it critically for important intellectual content. Both authors read and approved the final version.

## References

[B1] Warner KE, Martin EG (2003). The US tobacco control community's view of the future of tobacco harm reduction. Tob Control.

[B2] Broadstock EM (2007). Systematic review of the health effects of modified smokeless tobacco products.. New Zealand Health Technology Assessment Report.

[B3] Luo J, Ye W, Zendehdel K, Adami J, Adami HO, Boffetta P, Nyren O (2007). Oral use of Swedish moist snuff (snus) and risk for cancer of the mouth, lung, and pancreas in male construction workers: a retrospective cohort study. Lancet.

[B4] Rodu B, Godshall WT (2006). Tobacco harm reduction: an alternative cessation strategy for inveterate smokers. Harm Reduct J.

[B5] Levy DT, Mumford EA, Cummings KM, Gilpin EA, Giovino G, Hyland A, Sweanor D, Warner KE (2004). The relative risks of a low-nitrosamine smokeless tobacco product compared with smoking cigarettes: estimates of a panel of experts. Cancer Epidemiol Biomarkers Prev.

[B6] Foulds J, Ramstrom L, Burke M, Fagerstrom K (2003). Effect of smokeless tobacco (snus) on smoking and public health in Sweden. Tob Control.

[B7] Martinet Y, Bohadana A, Fagerstrom K (2007). Introducing oral tobacco for tobacco harm reduction: what are the main obstacles?. Harm Reduct J.

[B8] Savage L (2007). Experts fear Swedish snus sales in the U.S. could thwart anti-tobacco measures. J Natl Cancer Inst.

[B9] Gartner CE, Hall WD, Chapman S, Freeman B (2007). Should the health community promote smokeless tobacco (snus) as a harm reduction measure?. PLoS Med.

[B10] Nielsen AC (2007). Study finds 43 brands have billion dollar global presence.. http://www2.acnielsen.com/news/20011031.shtml.

[B11] London: RCP, Royal College of Physicians (2007). Harm Reduction in nicotine addiction: Helping people who can't quit. A report by the Tobacco Advisory Group of the Royal College of Physicians.

[B12] Holm H, Jarvis MJ, Russell MA, Feyerabend C (1992). Nicotine intake and dependence in Swedish snuff takers. Psychopharmacology (Berl).

[B13] Lunell E, Lunell M (2005). Steady-state nicotine plasma levels following use of four different types of Swedish snus compared with 2-mg Nicorette chewing gum: a crossover study. Nicotine Tob Res.

[B14] Ramstrom LM, Foulds J (2006). Role of snus in initiation and cessation of tobacco smoking in Sweden. Tob Control.

[B15] Gilljam H, Galanti MR (2003). Role of snus (oral moist snuff ) in smoking cessation and smoking reduction in Sweden. Addiction.

[B16] Furberg H, Bulik CL, Lerman C, Lichtenstein P, Pedersen NL, Sullivan P (2005). Is Swedish snus associated with smoking initiation or smoking cessation?. Tob Control.

[B17] Furberg H, Lichtenstein P, Pedersen NL, Bulik C, Sullivan PF (2006). Cigarettes and oral snuff use in Sweden: Prevalence and transitions. Addiction.

[B18] Stegmayr B, Eliasson M, Rodu B (2005). The decline of smoking in northern Sweden. Scand J Public Health.

[B19] Rodu B, Stegmayr B, Nasic S, Asplund K (2002). Impact of smokeless tobacco use on smoking in northern Sweden. J Intern Med.

[B20] Rodu B, Stegmayr B, Nasic S, Cole P, Asplund K (2003). Evolving patterns of tobacco use in northern Sweden. J Intern Med.

[B21] Fisher M (2007). Snus smokeless tobacco products. Presentation at the Life Sciences Research Office Meeting, October 3-4, 2007.

[B22] Foulds J, Stapleton J, Feyerabend C, Vesey C, Jarvis M, Russell MA (1992). Effect of transdermal nicotine patches on cigarette smoking: a double blind crossover study. Psychopharmacology (Berl).

[B23] Gartner CE, Hall WD, Vos T, Bertram MY, Wallace AL, Lim SS (2007). Assessment of Swedish snus for tobacco harm reduction: an epidemiological modelling study. Lancet.

[B24] Dunsby J, Bero L (2004). A nicotine delivery device without the nicotine? Tobacco industry development of low nicotine cigarettes. Tob Control.

[B25] Nelson L (2007). If you think snus is a safe alternative to smoking, think again. The Kansas City Star.

[B26] CDC (2007). Cigarette smoking among adults-United States, 2006.. MMWR CDC Surveillance Summaries.

[B27] Cummings KM, Brown A, O'Connor R (2007). The cigarette controversy. Cancer Epidemiol Biomarkers Prev.

[B28] Foulds J, Kozlowski L (2007). Snus--what should the public-health response be?. Lancet.

[B29] Kozlowski LT (2002). Harm reduction, public health, and human rights: smokers have a right to be informed of significant harm reduction options. Nicotine Tob Res.

